# Diagnosis of del(5q) MDS, 14 Years after JAK-2 Positive PV Appearance: Complete Remission of both Diseases with Lenalidomide Monotherapy

**DOI:** 10.4084/MJHID.2016.050

**Published:** 2016-10-20

**Authors:** Antonella Vaccarino, Irene Dogliotti, Fabio Marletto, Andrea Demarchi, Mario Bazzan

**Affiliations:** 1 Rare Diseases, Immunology, Immunohaematology and Haematology Department, San Giovanni Bosco Hospital, Turin, Italy; 2 Pathology Unit, San Giovanni Bosco Hospital, Turin, Italy

## Abstract

This is the report of the clinical case of a patient who presents the association of a JAK-2 positive chronic myeloproliferative neoplasia to a subsequent 5q- myelodysplastic syndrome, developed after about 14 years from the first diagnosis. Patient’s symptoms had rapidly worsened, and she became transfusion-dependent. Therapy with low-dose Lenalidomide quickly reduced the splenomegaly and completely brought white cells counts, haemoglobin, and platelets back to normal. After more than one year from the start, blood cell count is still normal. As far as we know, this is the first case of an effective treatment with Lenalidomide reported in this clinical setting.

## Case report

The JAK-2 V617F mutation has been found in almost all patients with polycythaemia vera, and in nearly 50% of those with primary myelofibrosis and with essential thrombocythaemia. However, the JAK-2 mutation may also occur in other nosological entities, such as neoplasms with overlapping dysplastic and myeloproliferative features. The concomitant presence of both the JAK-2 mutation and 5q- MDS has occasionally been reported, but little is known about disease characteristics and response to treatment and even less about the timing of these genetic abnormalities. Sokol et al. found that the two alterations were acquired by two different and independent clones.^1^

Ingram et al. analysed 97 MDS patients from 6 European countries for the JAK-2 V617F mutation and they detected its presence in 6/97 (6.2%) of the samples, thus identifying a cohort of patients with del(5q) syndrome and hypercellular marrow.^2^

Small subsets of del(5q) MDS were thus reported to have also developed the JAK-2 mutation.^2^,^3^ On the other hand, very few cases of MPN syndromes have been reported in which del(5q) occurred after the initial diagnosis.^4^,^5^,^6^,^7^,^8^,^9^,^10^ On the contrary, there are very few reports of patients with MPN syndromes in whom del(5q) took place after the initial diagnosis.^4^,^5^,^6^,^7^,^8^,^9^,^10^

## Case Report with Results

Herein we report the case of a 71 year old Caucasian female, whose the only known comorbidities were arterial hypertension and a beta-thalassemia trait. In 1998, at the age of 53, the patient first presented fatigue and diffuse pruritus; a complete blood count was performed which showed an increase in white blood cells, red cells and platelets. Polycythaemia Vera was then diagnosed in another hospital by a bone marrow biopsy during the same year and the patient received Hydroxyurea as first-line treatment. She first came to our Haematology Unit about 10 years later, i.e., in April 2008; at that time she was still taking hydroxyurea 500 mg daily, five days per week and acetylsalicylic acid 100 mg per day, together with bisoprolol; her blood count was stable, and kidney, liver, and thyroid functions were normal. Electrolytes, glycaemia, and cholesterol were all within normal ranges. On physical examination, we observed no enlargement of the superficial lymphnodes, of the liver or of the spleen.

**Figure 1:**
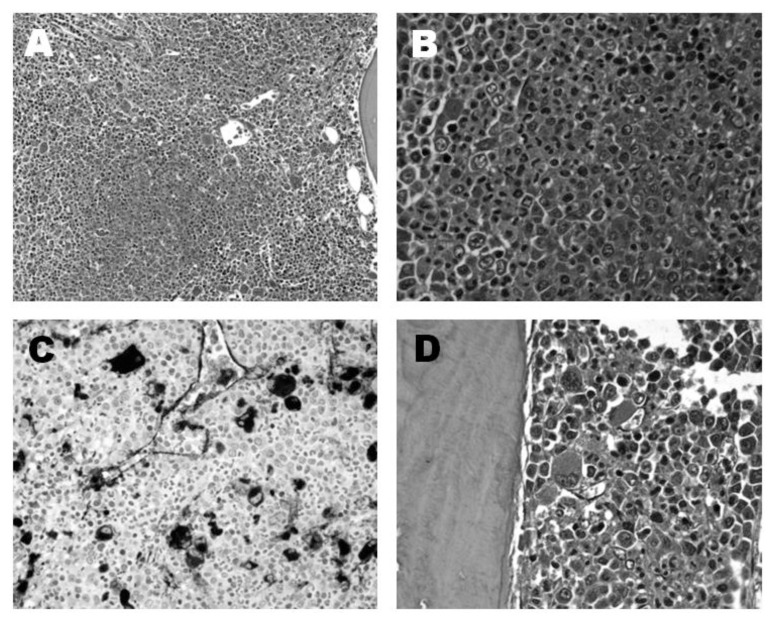
Light microscopy of a bone marrow biopsy of a patient with PV and 5q deletion. Light microscopy of a bone marrow biopsy showing: A) 100% marrow cellularity with dysplastic megakaryocytes and foci of immature precursors in abnormal localization (magnification: 100 HPF; staining: Hematoxylin-Eosin). B) Hypercellularity with evidence of myeloid hyperplasia (magnification: 630 HPF; staining: Hematoxylin-Eosin). C) CD 61 immunoperoxidase staining with intravasal haematopoiesis and focal clustering, very abnormal megakaryocytes (magnification: 400 HPF). D) Disorganized paratrabecular haematopoiesis with abnormal megakaryocytes (magnification: 630 HPF; staining: Hematoxylin-Eosin).

A search for both the JAK-2 V617F mutation and for the BCR/ABL mutation was performed, resulting in a heterozygous JAK-2 V617F mutation and the absence of a BCR/ABL mutation. The patient continued her ongoing therapy, and periodic follow-up visits showed no significant clinical changes until September 2011 when the spleen was palpable in deep inspiration: abdominal ultrasound showed a maximum spleen diameter of 14.6 cm. In March 2012, her white blood cell count rose to 33,290/mm^3^, platelets were 605,000/mm^3^, while haemoglobin was slightly decreased (11.3g/dL). In May 2012, on physical examination, her spleen and liver were respectively 2 and 1 cm below the costal margin. A few days later, haemoglobin levels decreased to 9.5g/dL, and the patient referred palpitations as the primary symptom. Four months later haemoglobin further dropped to 8.8 g/dL; LDH rose to 556, but white cells and platelets were stable. The patient underwent an abdominal CT scan which showed normal liver diameter and enlarged spleen (18 cm maximum diameter, with a uniform pattern). The bone marrow biopsy was repeated in October 2012: histopathological examination showed an evolution from Polycythaemia Vera to a Myelodysplastic/ Myeloproliferative syndrome, with a myeloblast percentage of 4% (Figure 1); bone marrow megakaryocytes had not the typical aspect of the del(5) syndrome. Pearl’s staining was performed and resulted negative. The monocytes count was not increased in the bone marrow and peripheral blood. In October 2012, complete karyotype analysis on her bone marrow showed 46, XX, del(5)(q13q32), del(13)(q14). White blood cells rose to 41,700/mm^3^, with absolute neutrophilia but in the absence of circulating myeloblasts; platelets were 242,000/mm^3^, haemoglobin remained at 8 g/dL, while the spleen, on physical examination, was found to reach the transverse umbilical line. The erythropoietin level before treatment was 125 mU /ml. Erythropoietin 40,000 U SC once a week was first started, then increased to twice a week, and red cells were transfused when haemoglobin dropped below 8 g/dL. Tests carried out in January 2013, showed a worsening of blood count values (Hb 7.7 g/dL, WBC 33,400/mm3, platelets 154,000/mm^3^). Moreover, there was also an increase in the need for transfusions (3 monthly units of RBCs), therefore, a first 21-day course of lenalidomide (Revlimid) 10 mg daily was started on 9^th^ February 2013. After the first lenalidomide course, the patient reported an improvement in her general conditions, despite mild oedema of the lower limbs; blood count showed that WBCs had dropped to 22,640/mm^3^, platelets were 266,000/mm^3^, and Hb was 7.7 g/dL thus requiring RBC transfusion. Hydroxyurea was discontinued, and low-dose hydrochlorothiazide and amiloride were associated to reduce the swelling of the legs. After the second course of lenalidomide, her blood count showed a dramatic improvement: WBCs dropped to 6,000/mm^3^ and haemoglobin rose to 9.9 g/dL, while platelets were stable (228,000/mm^3^). Erythropoietin treatment was discontinued. The spleen was palpable 1 cm above the transverse umbilical line and was significantly reduced in volume. Three months after the start of lenalidomide therapy, the patient’s blood count completely returned to normal with a haemoglobin level of 14 g/dL, while platelets and WBCs were steadily normal. Moreover, the spleen size reduced to 2 cm below the costal margin. After six courses, the patient reported recurrent abdominal pain and vomiting, together with diarrhoea, leading to hypotension and bradycardia, and she was admitted to the hospital. These GI tract symptoms remained for approximately three months, after which the therapy prescribed by the gastroenterlogist (PPIs, spasmolytics, symethicone, alginate, and probiotics) resulted effective. However, we decided to reduce the Lenalidomide dosage to 5 mg per day, after completing 10 courses. Once the dose of lenalidomide was lowered to 5 mg (November 2013), the GI symptoms disappeared, and the haematological response remained stable. To date, the patient has undergone eighteen cycles of lenalidomide. Her general conditions are fine, and she no longer has abdominal pain or diarrhoea. Her most recent blood test (July, 2015) showed a WBC count of 5,600/mm^3^ (neutrophil granulocytes 3,000/mm^3^), haemoglobin 12.4 g/dl, platelets 158,000/mm^3^ (Table 1). US scan showed that the spleen diameter was 14 cm. A cytogenetic examination on peripheral blood was repeated, and the del(5q) was no longer present, while the JAK-2 mutation still was, the allelic burden being 20.7%.

## Discussion

To date, few studies have analysed the association of myeloproliferative/myelodysplastic disorders and the presence of JAK-2 V617F mutation together with a 5q deletion; a large case series of these patients is currently lacking due to the relative rarity of the condition. The timing of appearance of the cytogenetic and molecular alterations was different, and most of the patients were diagnosed with myelodysplasia before the presence of the JAK-2 mutation was detected by molecular screening or due to the increase in myeloproliferative features. In fact, among myeloproliferative disorders, the 5q deletion has rarely been found, mostly in patients with primary myelofibrosis, and it is particularly rare in polycythaemia vera patients. Thus, it is difficult to determine whether the JAK-2 mutation represents a late event in an underlying myelodysplastic bone marrow, or rather an early event in the course of the disease; some authors have hypothesized that the two alterations may occur in two different and independent clones.^1^ Our clinical case seems to support this hypothesis since the myeloproliferative syndrome was diagnosed 14 years prior to the appearance of the 5q deletion. The patient received HU for many years; the hypothesis that she developed a HU-related MDS needs to be considered, but the evolution to acute myeloid leukemia or myelodysplasia (AML/MDS) is a part of the natural history of Polycythemia Vera. Data concerning the impact of HU on this evolution are not well established but this is theoretically possible.^11^

Lenalidomide therapy has proven to be efficacious for treating low risk and intermediate-1 risk myelodysplastic syndromes showing 5q deletion, with or without additional cytogenetic abnormalities; lenalidomide as a single agent therapy has led to an improvement in haemopoiesis and to a reduced need for transfusions in these patients. Little is known, however, about the mechanisms that lead to such clinical response, whether it is due to the direct cytotoxic effect shown *in vitro* or to an immunomodulatory and indirect cytotoxic effect. Only sporadic cases have been reported involving the administration of lenalidomide to patients having an association of the JAK-2 V617F mutation and the 5q deletion without additional cytogenetic anomalies; one of them proved to be refractory to lenalidomide treatment,^8^ while two patients showed a good response to it.^3^,^10^

In our case, the pathogenesis of splenomegaly was most likely related to the progression of myeloproliferative neoplasia. Considering the good response to lenalidomide treatment, we cannot rule out that splenomegaly may be linked, at least in part, to the infiltration of the del(5q) clone.

In our opinion, this is an interesting clinical case in which monotherapy with low dose lenalidomide proved to be effective in achieving sustainable clinical, haematological and molecular remission. Regular, non-dysplastic hemopoiesis was restored, our patient was no longer transfusion-dependent, splenomegaly decreased and the del(5q) mutation disappeared in the peripheral blood. More studies are needed to clarify the biological effects of lenalidomide on patients showing myelodysplastic/myeloproliferative features. It is important to consider lenalidomide as an option for treating selected patients with 5q- syndrome and proliferative bone marrow since it proved, in some cases, to be effective as a single agent and to have quite good subjective tolerance.

## Figures and Tables

**Table 1:**
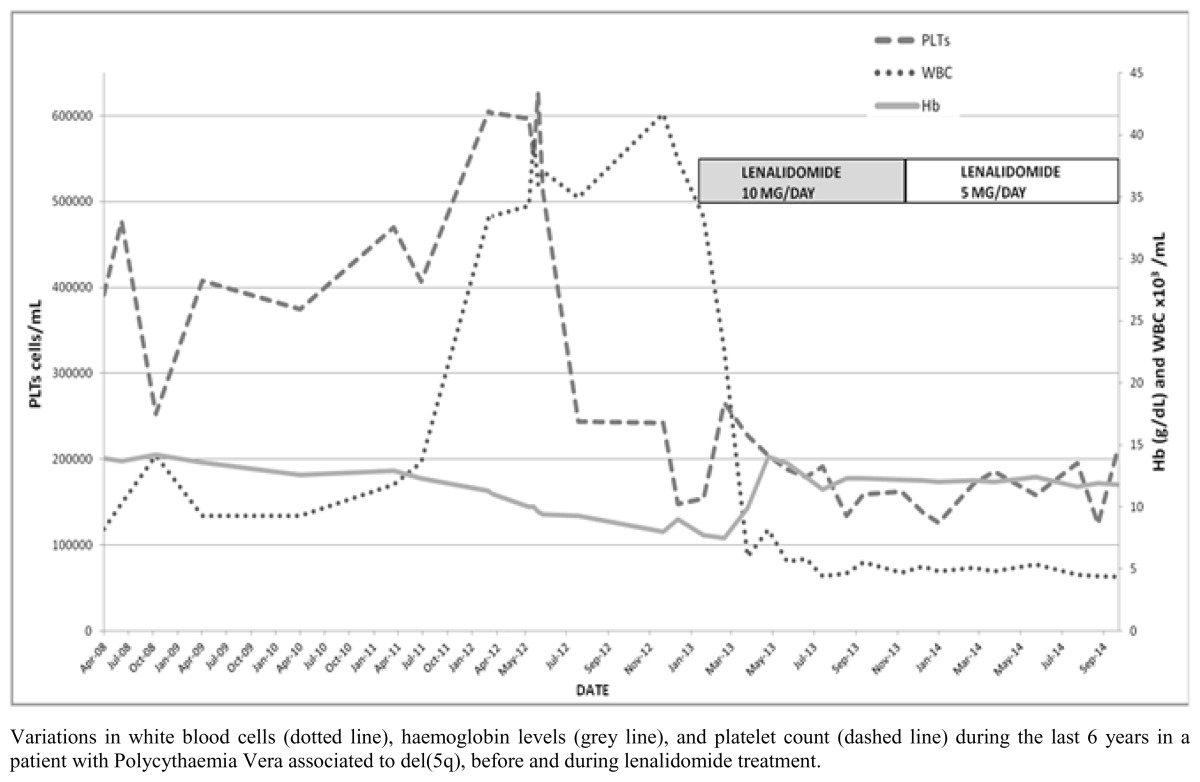
Blood Count trend before and during lenalidomide treatment.
